# Volatile Anesthetic Sevoflurane Precursor 1,1,1,3,3,3-Hexafluoro-2-Propanol (HFIP) Exerts an Anti-Prion Activity in Prion-Infected Culture Cells

**DOI:** 10.1007/s11064-021-03344-8

**Published:** 2021-05-27

**Authors:** Takuto Shimizu, Emiko Nogami, Yuka Ito, Kazuo Morikawa, Masaki Nagane, Tadashi Yamashita, Tsuyoshi Ogawa, Fuyuki Kametani, Hisashi Yagi, Naomi Hachiya

**Affiliations:** 1 Laboratory of Biochemistry, School of Veterinary Medicine, Azabu University, 1-17-71 Fuchinobe, Chuo-ku, Sagamihara-shi, Kanagawa 252-5201 Japan; 2 Chemical Research Center, Central Glass Co., Ltd., 17-5, Nakadai 2, Kawagoe City, Saitama 350-1159 Japan; 3 Faculty of Engineering, Tottori University, 4-101 Koyama-Minami, Tottori, 680-8552 Japan; 4 Center for Research on Green Sustainable Chemistry, Tottori University, 4-101 Koyama-Minami, Tottori, 680-8552 Japan; 5 Tokyo Metropolitan Industrial Technology Research Institute, 2-4-10 Aomi, Koto-Ku, Tokyo, 135-0064 Japan; 6 Chemicals Business Development Department, Central Glass Co., Ltd, Kowa-Hitotsubashi Building, 7-1 Kanda-Nishikicho 3, Chiyoda-ku, Tokyo 101-0054 Japan; 7 Tokyo Metropolitan Institute of Medical Science, 2-1-6 Kamikitazawa, Setagaya-Ku, Tokyo 156-8506 Japan

**Keywords:** Prion, Prion disease, Prion protein, Anti-prion drug, Hexafluoro isopropanol, Amyloid, Amyloid-beta (Aβ), Neurodegeneration, Neurodegenerative disease

## Abstract

Prion disease is a neurodegenerative disorder with progressive neurologic symptoms and accelerated cognitive decline. The causative protein of prion disease is the prion protein (PrP), and structural transition of PrP from the normal helix rich form (PrP^C^) to the abnormal β-sheet rich form (PrP^Sc^) occurs in prion disease. While so far numerous therapeutic agents for prion diseases have been developed, none of them are still useful. A fluorinated alcohol, hexafluoro isopropanol (HFIP), is a precursor to the inhalational anesthetic sevoflurane and its metabolites. HFIP is also known as a robust α-helix inducer and is widely used as a solvent for highly aggregated peptides. Here we show that the α-helix-inducing activity of HFIP caused the conformational transformation of the fibrous structure of PrP into amorphous aggregates in vitro. HFIP added to the ScN2a cell medium, which continuously expresses PrP^Sc^, reduced PrP^Sc^ protease resistance after 24-h incubation. It was also clarified that ScN2a cells are more susceptible to HFIP than any of the cells being compared. Based on these findings, HFIP is expected to develop as a therapeutic agent for prion disease.

## Introduction

Prions are infectious pathogens that cause fatal neurodegenerative diseases by altering the three-dimensional structure of the causative protein by yet unknown mechanism. The protein that causes prion disease is the prion protein (PrP); PrP undergoes structural conversion from a normal form of α-helix-rich PrP^C^ to an abnormal form β-sheet-rich PrP^Sc^ although there is no difference of amino acid sequence between them [1]. There are three types of human prion disease: idiopathic/sporadic, hereditary, and infectious. The most common type is idiopathic/sporadic, accounting for around 80% of all prion diseases [2]. Approximately 15% are inherited with several mutations in the open reading frame of the PrP gene, and mutations in hereditary prion disease raise the risk of a structural change in PrP [3]. Structural changes in PrP due to extrinsic PrP^Sc^ occur in infectious prion disease, including variant CJD (vCJD) [4]. vCJD, thought to be caused by eating prion-affected beef, occur in relatively young patients and are characterized by the presence of florid plaques, especially in the cerebral and cerebellar cortices. Interestingly, the NMR study also revealed that the conformation of PrP in bovine and humans is essentially the same [5]. Methionine homozygosity (MM) at codon 129 of the prion protein encoding gene PRNP has been observed in vCJD patients. In addition, vCJD in 129MV and 129VV patients has a long incubation period and is a concern for secondary infection [6].

PrP is a protein belonging to the secretory pathway with an endoplasmic reticulum translocation signal at the amino terminus and a glycosylphosphatidylinositol (GPI) additional signal at the carboxyl terminus for lipid raft targeting [7]. We have previously reported that endogenous PrP is localized in the cell membrane and microtubules and moves intracellularly [8, 9]. PrP is also present in mitochondria [10–12] and we have recently discovered that the 18 amino acids in the residues 122–139 of PrP are a cryptic mitochondrial targeting signal of PrP [13]. Despite the many attempts to elucidate the physiological role of PrP, it is still not well understood [14, 15].

Sevoflurane is a commonly used inhalation anesthetic with relatively fewer side effects than other inhalation anesthetics because it is not metabolized to acyl halides [16, 17]. The fluorinated alcohol 1,1,1,1,3,3,3-hexafluoro-2-propanol (HFIP) is a precursor of sevoflurane. Metabolized HFIP is excreted in the urine as HFIP-glucuronide after phase I oxidation reaction by cytochrome P-450 2E1 (CYP2E1) and phase II glucuronidation reaction by UDP-glucuronosyltransferase [18, 19].

No toxicity for HFIP at clinically derived concentrations has been reported to date. Besides, HFIP suppresses endotoxin-stimulated inflammatory mediator secretion and improves the survival rate of septic peritonitis mice models [20]. Fluorinated alcohols, such as HFIP, have a particularly strong protein-denaturing activity and break the β-sheet structure leading to α-helix. Therefore, it has been used as a solvent to dissolve peptide aggregates such as amyloid β (Aβ) peptides [21–24].

PrP^Sc^ is thought to consist of many structures rather than of a uniform structure. As a therapeutic agent for prion diseases, attempts have also been made to identify compounds that inhibit the structural conversion of PrP^C^ to PrP^Sc^, however many of these drugs have resulted in drug resistant prion strains [25]. On the other hand, HFIP has strong α-helix-inducing activity, should be able to unwind PrP^Sc^ into PrP^C^, and PrP^Sc^ structure itself will be normalized. If this is the case, issues about the development of PrP^Sc^ drug resistant strains can be eliminated.

In this study, we explored the possibility of HFIP as a therapeutic drug for prion diseases using recombinant PrP and scrapie-infected mouse neuroblastoma cells, an established in vitro model of prion disease.

## Results

**Fig. 1:**
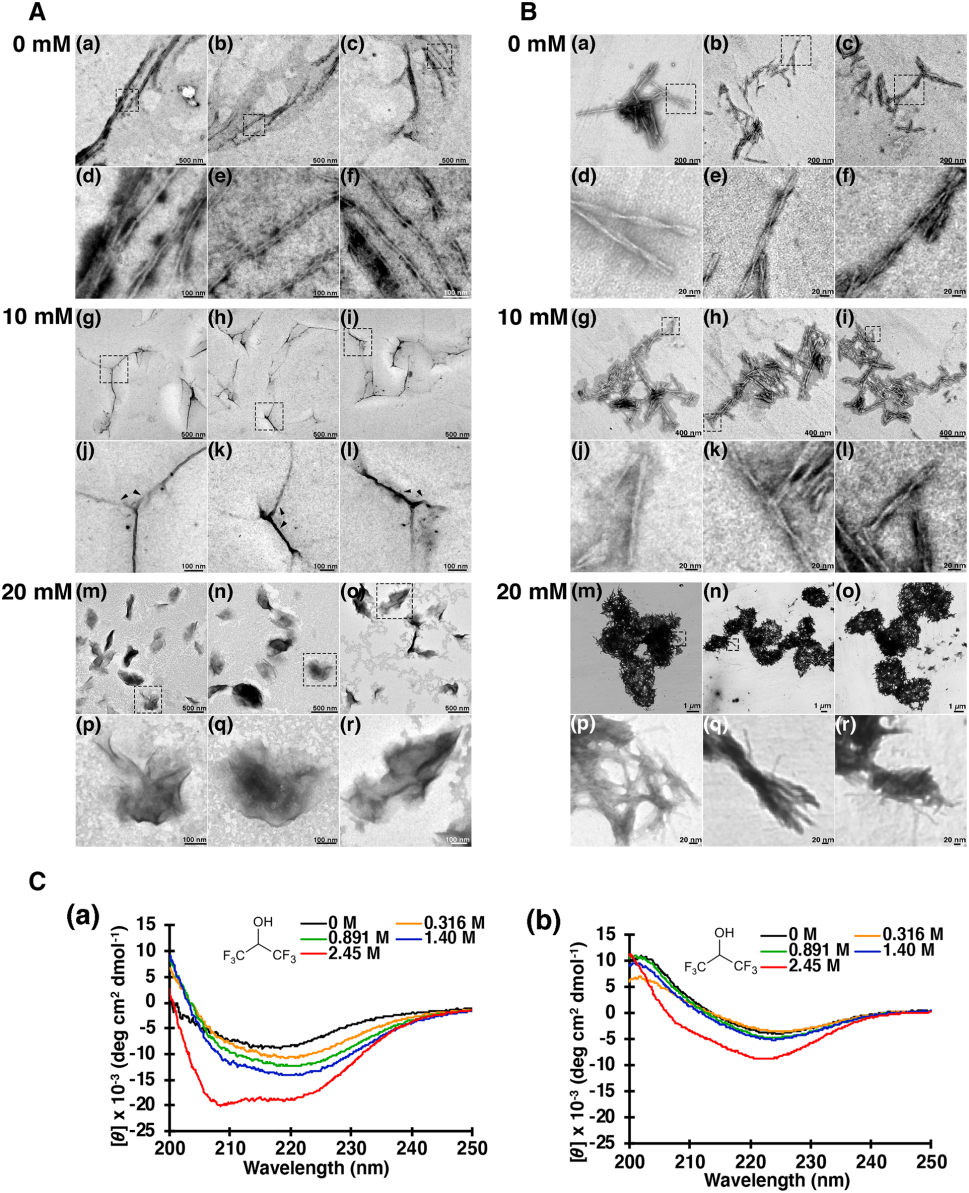
HFIP has a different effect on PrP and Aβ (1–40). **A** Ultrastructure of PrP fibers. When recombinant PrP was incubated with PBS in the absence of HFIP for 24 h, PrP fibrillated and formed an unbranched linear structure (**A** (*a*)–(*c*), magnified images (*d*)–(*f*)). After the incubation with 10 mM HFIP for 24 h at 37 °C, the PrP fibrils were transformed into three-pronged structures. (**A** (*g*)–(*i*), magnified images (*j*)–(*l*), Arrowheads). Only amorphous aggregates have been found in 20 mM of HFIP (**A** (*m*)–(*o*), magnified images (*p*)–(*r*)). The scale bar for each panel is shown in the figure. **B** Ultrastructure of the amyloid fibrils of Aβ (1–40). The fibrous structure of Aβ (1–40) did not alter with the 24-h incubation of 10 and 20 mM of HFIP. Instead, amyloid fibrils have been associated in a concentration-dependent manner with HFIP. **C** CD measurement of PrP and Aβ (1–40) amyloid. The secondary structure of PrP modified with the HFIP concentration (*a*). Aβ (1–40) amyloid is resistant to HFIP and is unlikely to undergo secondary structural changes (*b*)

The effect of HFIP to PrP fibrils produced by the recombinant PrP was observed using transmission electron microscopy (TEM). When recombinant PrP was incubated with PBS in the absence of HFIP for 24 h, PrP fibrillated and formed an unbranched linear structure (Fig. 1A (a)–(c), magnified images (d)–(f)). When the PrP fibrils were incubated with 10 mM HFIP for 24 h at 37 °C, the linear PrP fibrils changed into three-pronged structures (Fig. 1A (g)–(i), magnified images (j)–(l), Arrowheads). Moreover, 20 mM of HFIP completely transformed the fibrous PrP into an amorphous shape (Fig. 1 A (m)–(o), magnified images (p)–(r)), indicating that HFIP induces a drastic conformation-changing activity to fibrous PrP. Compared to PrP, the Aβ (1–40) (Fig. 1B (a)–(f)) did not alter the fibrous structure in the presence of HFIP (Fig. 1B (g)–(r)), but a rather enhanced association of the amyloid fibrils. In addition, CD spectroscopy was used to measure changes in the secondary structure of PrP and Aβ (1–40) after HFIP treatment. Since CD spectroscopy makes it difficult to evaluate at lower HFIP concentrations than in other experimental conditions, we used higher concentrations of HFIP and substrates. The β-sheet structure of PrP has modified to α-helix-rich HFIP-dependent structure (Fig. 1C (a)) while the Aβ (1–40) structure has remained unchanged up to 2.45 M HFIP (Fig. 1C (b)). These findings indicate that HFIP has a distinct effect on PrP and Aβ (1–40).

**Fig. 2:**
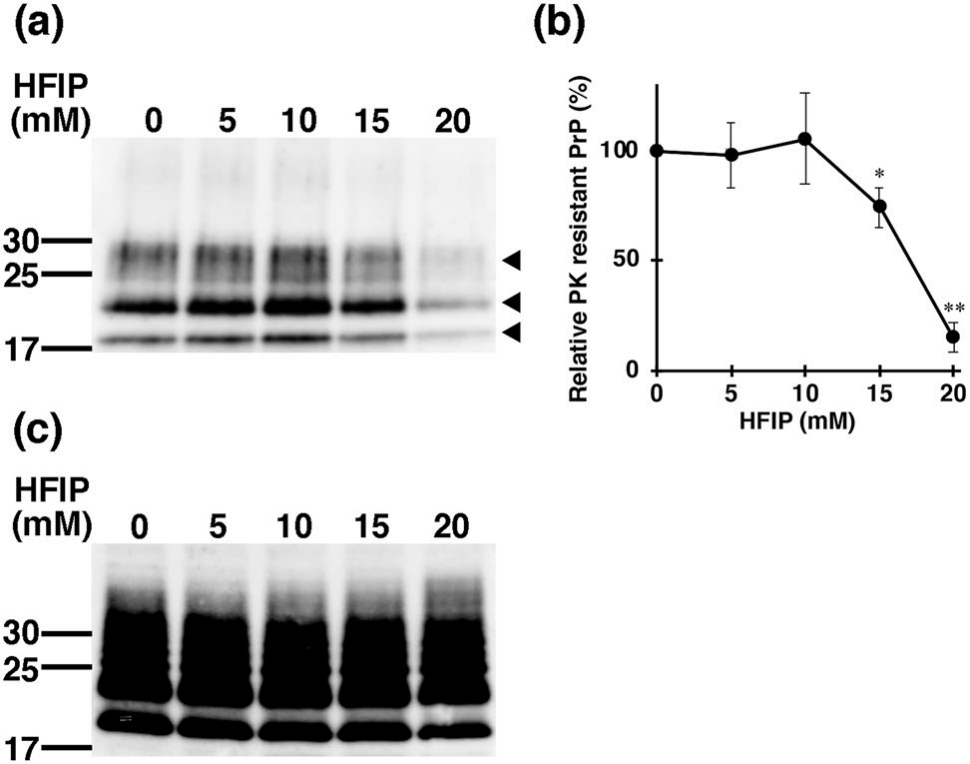
HFIP increases the PK sensitivity of ScN2a cells. HFIP was added to the medium of ScN2a cells as follows; 0, 5, 10, 15 and 20 mM. After culturing cells for 24 h, PK resistance was detected as in Materials and Methods. **a** Western blotting of PK-resistant bands (The unglycosylated, mono and diglycosylated PrP forms. Arrowheads.) of PrP. The amount of protein used in this assay was 100 µg/tube. **b** Quantitative result. The statistically significant difference was shown by *p < 0.05, **p < 0.01 for 0 mM HFIP. **c** Samples without PK treatment were subjected to SDS-PAGE and Western blotting with an anti-PrP antibody

PrP^C^ is easily digested by the proteolytic enzyme proteinase K (PK), while PrP^Sc^ exhibits partial PK resistance. PrP^Sc^ formation inhibition is a major target for therapeutic intervention in prion disease; thus, ScN2a cells continuously producing PrP^Sc^ are valuable models for detecting anti-prion activity [26]. PK resistance of PrP^Sc^ did not change with the incubation at 5 or 10 mM of HFIP for 24 h; however, HFIP at 15 mM decreased PK resistance of PrP^Sc^ by approximately 40% compared to the control. When the HFIP concentration increased to 20 mM, the PK resistance bands of PrP^Sc^ significantly decreased (Fig. 2).

**Fig. 3:**
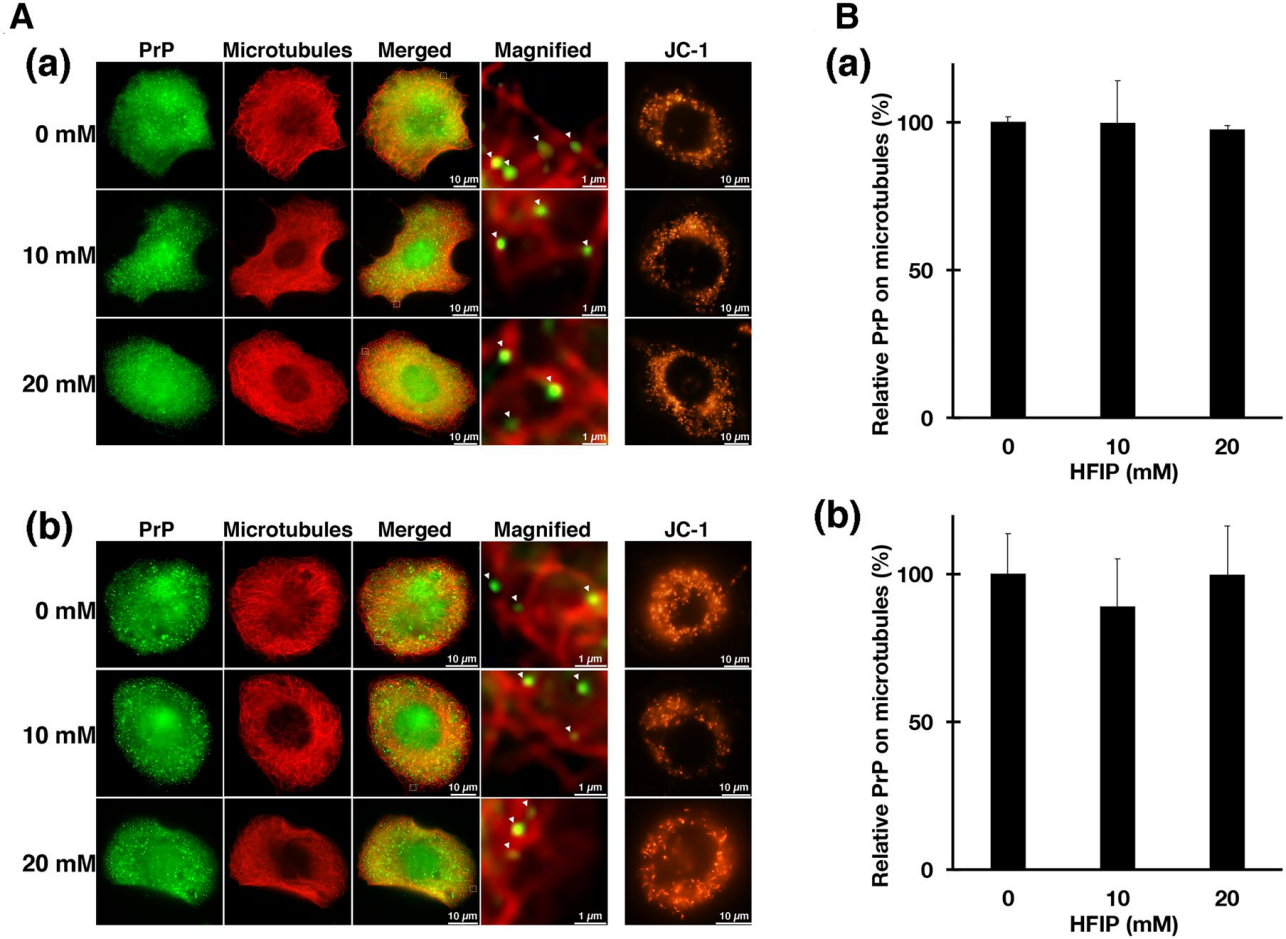
Subcellular localization of endogenous PrP in the presence of HFIP and mitochondrial membrane potential. **A** To identify endogenous PrP in ScN2a (*a*) and N2a (*b*) cells, anti-PrP peptide antibody (PrP 76–90), which recognizes the N-terminal region of PrP, was used. An anti-tubulin antibody was used to detect microtubules. Arrowheads indicate PrP (green) on microtubules (red). JC-1 staining revealed that mitochondrial membrane potential was maintained normally even in the presence of HFIP. **B** The number of PrPs on microtubules was counted for fluorescent immunostaining. The number of PrPs located on microtubules per unit area (3 μm/each) was counted. The relative percent value was set at 100% of the amount of PrPs found on microtubules in the absence of HFIP. The number of PrPs found on microtubules in the presence of HFIP has been counted and measured. The bars in the figure represent the standard error

If a structural change in PrP^Sc^ decreases PK resistance in ScN2a cells, it should be recognized by various intracellular chaperones. In that case, the protein quality control system should be triggered, causing changes in the subcellular distribution of PrPs [27]. Therefore, we used indirect immunofluorescence antibody technique to investigate the localization change of endogenous PrP in the presence of HFIP (Fig. 3A).

Endogenous PrP is localized on microtubules. As with other cargo proteins, PrP does not bind directly to microtubules but binds to motor and adaptor proteins for anterograde and retrograde transport [8, 9]. At this time, the angle at which a cargo molecule, such as PrP, binds to microtubules through an adaptor molecule is not always constant and can take on a 360-degree configuration from microtubules. As a result, some PrPs on microtubules merge yellow while others do not, such as when PrPs are 90-degrees away from the microtubules. Even under such circumstances, PrPs in Fig. 3A were sufficiently close to the microtubules, and some yellowish merged foci were found in the magnified images, so we concluded that HFIP had no effect on PrP subcellular localization or association with microtubules (ScN2a; Fig. 3A(a), Fig. 3B(a), N2a; Fig. 3B(b)).

Maintaining the membrane potential of mitochondria ensures that normal energy production is being performed and indicates that cells exhibit a healthy state. JC-1 dye accumulates at the mitochondrial membrane in a potential-dependent manner: when the membrane potential is maintained, aggregated JC-1 fluoresces red, but when the membrane potential decreases, JC-1 remains monomeric and emits green fluorescence. Since HFIP did not affect the membrane potential of mitochondria in both cells (ScN2a; Fig. 3A (a) (JC-1), N2a; Fig. 3A (b) (JC-1)), the intracellular effect of the addition of HFIP was, if any, extremely minimal.

**Fig. 4:**
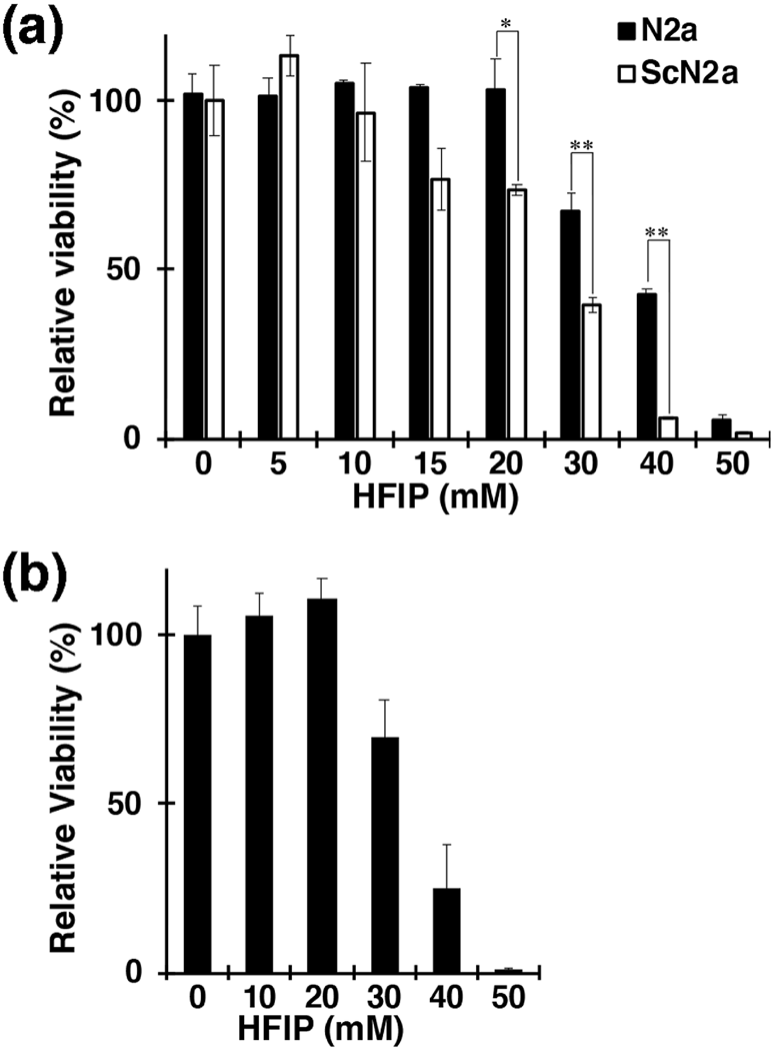
Cytotoxicity of HFIP. ScN2a cells were susceptible to HFIP. After adding HFIP at the concentration shown in the figure to the medium of N2a (**a**), ScN2a (**a**) and COS-7 (**b**) cells, cells were cultured for 24 h then stained with crystal violet to count the viable cells. Bars in the graph indicate the SD. The statistically significant difference in panel (**a**) was shown by *p < 0.05 **p < 0.01 for N2a cells

Finally, the sensitivity of the cultured cells to HFIP was examined by crystal violet staining. As shown in Fig. 4(a), in N2a cells, HFIP did not affect the cell viability up to a concentration of 20 mM. The susceptibility of non-neuronal cells to HFIP was also examined in the same way using COS-7 cells, which are fibroblasts, and they were resistant up to a concentration of 20 mM, similar to N2a cells (Fig. 4(b)). On the other hand, the viability of ScN2a cells was reduced by about 30% compared to N2a cells at a HFIP concentration of 15 mM. At a HFIP concentration of 40 mM, most of the ScN2a cells failed to survive, while N2a cells could grow by about 40%. Therefore, it was suggested that ScN2a cells were more susceptible to HFIP.

## Discussions

Persistent prion infected ScN2a cells have been commonly used as a pre-screening step to discover new anti-prion agents. To date, an assay system using ScN2a cells has been used to classify anti-prion agents such as antibodies [28], dominant-negative molecules [29, 30] and low molecular weight compounds [31]. However, despite so many attempts, no successful cure and clinically beneficial drugs for prion disease have been found.

Fluorinated alcohols, such as HFIP, are unique alcohols in which fluorine is bound to carbon rather than hydrogen while sharing a similar overall structure with 'natural' alcohols. Since the electronegativity of the fluorine molecule is strong, it destabilizes the hydrophobic interaction of the substrate molecules and facilitates the bonding of intramolecular hydrogen. This activity is thought to help HFIP break the β-sheet structure and induce α-helical conformation; thus, HFIP is also used as a solvent for Aβ peptides with a high aggregate propensity [23, 24].

In a previous study, Wille et al*.* reported that the treatment of prion rods made from scrapie-infected Syrian hamster brain extract with HFIP had modified their structure to flat ribbon-shaped [32]. In this regard, we have found that the incubation of PrP fibrils consisted of recombinant protein with HFIP has modified the structure into amorphous aggregates (Fig. 1A).

Notably, the helix-inducing activity of HFIP was reported only at high HFIP concentrations in previous studies. For example, when Aβ (11–28) containing the core region of the Aβ aggregation was used as a substrate, the helix structure was seen in a mixture of the 90% HFIP and 10% water in CD measurements, while the water content increased to 90%, the β-sheet content has increased [22]. Furthermore, low concentrations of HFIP have also been reported to increase the formation of Aβ fibril [33, 34].

10 mM (0.17%) or 20 mM (0.34%) of HFIP were used in our experiments and thus Aβ (1–40) fibrils did not alter the conformation and induced the association of fibrils (Fig. 1B). The difference in the effect of HFIP on PrP and Aβ amyloid fibrils at these concentrations may be due to the different construction of each fibril. Aβ (1–40) fibrils form a tight cross-β structure, with almost 90% of the molecule being the backbone [35, 36]. Thus, HFIP is presumably unable to reach the inside of the molecule and stays on the surface of the fibrils, which is thought to promote the association of the fibers. Whereas PrP consists of a non-structural region, a three-helical structure, and a domain structure consisting of two antiparallel β-chains [37, 38]. In addition, Wang et al*.* recently reported that PrP fibers with recombinant full-length human PrP^C^ (residues 23–231) made up of two protofibrils entwined in the left-handed helix using cryo-electron microscopy [39]. As a result, PrP fibrils can easily undergo more alteration, allowing HFIP to enter the inside of the molecule and transfer the structure a fibrous to amorphous.

PrP^Sc^ accumulates in the cell membrane, and the PrP^C^-to-PrP^Sc^ conversion process is thought to occur primarily in cell membrane lipid rafts or extracellularly [40]. If HFIP induces structural modification of PrP^Sc^ within ScN2a cells that alters the PK sensitivity (Fig. 2), intracellular molecular chaperones may have been recognized and their protein quality control system will be triggered. Consequently, the intracellular distribution of PrP will be changed [41]. However, this was not the case in our experimental findings (Fig. 3); thus, HFIP may not reached the inside of the cell, and it is more appropriate to think that the decrease in PK resistance due to the structural change of PrP^Sc^ occurred on the cell membrane. Of note, previous screening of anti-prion activity using ScN2a cells required long incubation for periods of days [42–44] to confirm the anti-prion effect. In view of this, it is remarkable that HFIP was successful in a 24 h treatment.

The concentration range of inhibitory HFIP activity of PrP^Sc^ formation in ScN2a cells is between 15 and 20 mM, as shown in Fig. 2. On the other hand, the cytotoxicity of HFIP increased significantly from 20 mM onwards, as shown in Fig. 3. The therapeutic window of HFIP for ScN2a cells, therefore, appears to be very small. Besides, the α-helix-inducing activity of HFIP is non-specific, which may cause serious side effects. Thus, it is difficult to immediately demonstrate HFIP itself as a therapeutic agent for prion disease. To solve these problems, it is important to synthesize HFIP derivatives in which the concentration that causes cytotoxicity and the concentration that causes α-helix-inducing activity are different. Furthermore, it is also important that the HFIP derivative has substrate specificity for PrP and that the α-helix-inducing activity of HFIP does not function non-specifically.

Lipid rafts are highly active, short-lived microdomains found in cholesterol- and sphingolipid-rich cell membranes. It is also involved as a platform for important cellular pathways such as membrane transport, signal transduction, and immune response activation [45]. The abundance and efficacy of these microdomains are highly dependent on the availability of cholesterol [46, 47]. Biosynthesis of cholesterol has been reported to be enhanced by prion infection [48], suggesting a link between the metabolism of cholesterol and the transmission of prion. Cholesterol is also required for PrP cell surface expression and stabilization [49], and PrP^Sc^ with GPI anchors is present in lipid rafts [50]; thus, cholesterol depletion inhibits prion replication by inhibiting the transport of PrP to lipid rafts [51, 52].

In general, alcohol has the activity of depleting cholesterol from the target cell membrane and can increase the membrane's fluidity. Fluoride alcohols have been reported to cause lipid bilayer leaks, reduce lipid acyl chain order, alter the temperature of transition phase lipids [53], and induce micellar aggregation [54]. Low concentrations of HFIP (1–3 mM) are known to influence the ion permeability of membrane proteins such as Kv1.3 K^+^ channels [55] and gramicidin channels [53]. Therefore, we cannot rule out the possibility that HFIP may have destabilized lipids in our experiments and caused a reduction in PK tolerance in PrP^Sc^. It will be necessary to develop HFIP derivatives that do not affect lipids or display off-target effects to address this issue.

ScN2a cells are highly susceptible to HFIP, and approximately 40% of the cells died in the presence of 20 mM of HFIP. Whereas the same condition did not significantly kill N2a and COS-7 cells. One possible reason for the disparity in HFIP-susceptibility of ScN2a cells may be that the cell membranes' lipid composition in these cells might not be the same. Consequently, there could be a difference in sensitivity to HFIP for the reasons described above.

Interestingly, the mitochondrial membrane potential in ScN2a cells, which survived 20 mM HFIP, was normal even in the presence of PrP^Sc^ (Fig. 3A). The localization of endogenous PrP was also normal, indicating healthy state. Considering that ScN2a cells are more sensitive to HFIP than N2a cells (Fig. 4A), this result also suggests that treatment of ScN2a cells with HFIP eliminates HFIP-sensitive cell populations and allows only healthy cells. Furthermore, a small molecule such as HFIP (molecular weight 168.04) can be useful as a therapeutic agent for neurodegenerative diseases because it can easily cross the blood–brain barrier, which is typically permeable to molecules with molecular weights below 400 [56]. Therefore, the pharmacokinetics of HFIP, a small molecule compound, may be beneficial.

As shown in previous research on the development of therapeutic agents for prion disease, the experimental approach using cultured cells is a very simplified model compared to the in vivo situation. In addition to cell culture experiments, it is essential to conduct animal experiments in the future.

## Materials and Methods

### Cell Culture

Mouse neuroblastoma Neuro2a (N2a) and COS-7 cells were obtained from the American Type Culture Collection. ScN2a cells were kindly supplied by Dr. Horiuchi (Hokkaido University, Japan). N2a cells and ScN2a cells were grown in Eagle’s Minimum Essential Medium, COS-7 cells were grown in Dulbecco’s modified Eagle’s medium containing 100 units/ml of penicillin and 100 µg/ml of streptomycin complemented by 10% of fetal bovine serum and 5% of CO_2_ in the humid incubator at 37ºC.

### Recombinant PrPs

The recombinant full-length hamster PrP (23–231) used for TEM observation was obtained from Thermo Fisher Scientific Prionics AG (Schlieren, Switzerland) and dissolved in PBS at a concentration of 1 µg/µl. Since the measurement of CD spectra requires a larger amount of PrP, *E. coli* was used to express mouse recombinant PrP for the measurement. Briefly, the full-length mouse PrP (23–230) expression vector encoding amino acid residues 23–230 of mouse PrP [30] was transfected into the *E. coli* BL21 (DE3) strain (Merck KGaA, Darmstadt, Germany) and pre-cultured in LB Broth (Invitrogen) at 37 °C for 18 h. *E. coli* was further inoculated in Terrific Broth (Invitrogen) containing 0.4% Glycerol at 37 °C for 3 h. Isopropyl-β-D-thiogalactopyranoside was added to the induction of expression for PrP at the final concentration of 500 µM. PrP was accumulated in *E. coli* as inclusion bodies. After the solubilization of the inclusion bodies with solubilization buffer (50 mM Tris–HCl (pH 7.5), 8 M Urea, 150 mM NaCl, 500 µM PMSF), the sample was centrifuged, and the resulting supernatant was applied to a Ni-Sepharose column (Cytiva, Tokyo, Japan) for the purification of PrP using the octapeptide repeat region of PrP has an affinity for nickel ions.

### In Vitro Fibrillogenesis of PrP and Aβ (1–40)

Hamster recombinant PrP used for TEM observation and mouse recombinant PrP used for CD spectrum measurement were dissolved in PBS and 5 mM Tris–HCl (pH 7.5), respectively, and allowed to form fibers by standing. Aβ-peptide (Human,1–40) (HCl Form) (Peptide Institute, Osaka, Japan) was dissolved in 0.05% ammonia water (Fujifilm Wako, Osaka, Japan) to 500 µM. Aβ (1–40) fibrils used for TEM observation were diluted by adding 50 mM sodium phosphate (pH 7.5) to a concentration of 100 µM. 150 µl of the solution was added to a 2 ml tube (Eppendorf AG, Hamburg, Germany) and shaken at 1,500 rpm for 16 h at room temperature using a MicroMixer E-36 (TAITEC CORPORATION, Saitama, Japan) to form fibers. For the samples used for CD spectra, Aβ (1–40) was diluted to 100 µM using PBS, and then 300 µl of the solution was added to a centrifuge tube. The sample was shaken at 250 rpm for 12 h at 37 °C using a BR-23FP (TAITEC CORPORATION, Saitama, Japan).

### Ultrastructure of PrP and Aβ (1–40)

The recombinant PrP and Aβ (1–40) were suspended at 14.6 µM then incubated with HFIP (Central Glass Co., Ltd. Tokyo, Japan) at 37 °C for 24 h, followed by glutaraldehyde fixation. 3 µl of specimens were added to the formvar-carbon-coated grids. The same volume of 1% (w/v) Potassium Eu-encapsulated Preyssler-type Phosphotungstate (FUJIFILM Wako Pure Chemical Co. Osaka, Japan) was applied to the grid. Specimens were observed by Titan Cubed G2 60-300 (FEI Group, Hillsboro, Oregon, USA).

### Circular Dichroism (CD) assay

The mouse recombinant PrP was dialyzed in 5 mM of Tris–HCl (pH 7.5), treated with HFIP at room temperature for 3 h at the concentration shown in the figure, then CD measurement (J-820, JASCO Corporation, Tokyo, Japan) was performed. Before the CD measurement, Aβ (1–40) was diluted in PBS, followed by shaking at 250 rpm (TAITEC BioShaker BR-23FP), at 37 °C for 12 h.

### PK-Resistance Assay

ScN2a cells were seeded on 6-well plates (1 × 10^6^ per well), cultured overnight, and replaced with a medium containing HFIP (0–20 mM) then incubated for 24 h. 500 µl of RIPA Buffer (FUJIFILM Wako Pure Chemical Co. Osaka, Japan) per well was added and incubated on ice for 1 min. The solubilized sample was centrifuged at 850 × *g* at 4 °C for 10 min, and the solubilized supernatant was collected. Protein concentration in the supernatant was quantified using the Total Protein Quantitation Kit, Bradford Ultra (Novexin Ltd., Cambridge, UK) according to the instruction manual. 100 µg of total protein were treated with 10 µg/mL PK at 37 °C for 30 min. Protease inhibitor cocktail (Nacalai Tesque, INC. Kyoto, Japan) was added to stop the PK activity and incubated at room temperature for 5 min then centrifuged at 20,000 × *g* at 4 °C for 20 min. The sedimented fraction was collected as PK-resistant.

### Western Blotting

Western blotting was performed as previously reported [13]. The membrane was examined with an anti-PrP antibody SAF83 (Bertin Bioreagent. Montigny le Bretonneux, France) diluted with PBS-T (1:1000) for overnight at room temperature as the first antibody then incubated with HRP-conjugated anti-mouse IgGs (H + L) (Promega Co. Madison, WI, USA) as a secondary antibody (1:5000).

### Immunofluorescent Microscopy Observation

The immunofluorescent antibody method was performed as previously reported [8]. Briefly, the fixed cells were incubated overnight at 4ºC with anti-PrP peptide (PrP 76–90) antibody (1: 200) and α-tubulin antibody (1: 500) (Sigma-Aldrich. St. Louis, MO, USA). Alexa Fluor® Plus 488-conjugated anti-rabbit IgG (1:200) and Alexa Fluor® 594-conjugated anti-mouse IgG (1:1000) were used as a secondary antibody. Fluorescent images were acquired using a microscope IX73 (Olympus Corp., Tokyo, Japan).

### JC-1 Staining

JC-1 (FUJIFILM Wako Pure Chemical Co. Osaka, Japan) was added to the culture medium of N2a and ScN2a cells with a final concentration of 5 µg/ml and incubated at 37 °C for 30 min. After the incubation, cells were washed and replaced with phenol red-free L-15 medium (Thermo Fisher Scientific, Waltham, MA, USA). Fluorescence images were acquired using an microscope IX73 (Olympus Corp., Tokyo, Japan).

### Crystal Violet Staining

The susceptibility of HFIP in cells was evaluated by crystal violet staining. Cells (1 × 10^6^ per well) were seeded onto 6-well plates and cultured overnight; then, the medium was replaced with HFIP-containing medium (0–50 mM). After treatment with HFIP, cells were fixed with 100% methanol for 30 min then stained with 0.05% crystal violet (FUJIFILM Wako Pure Chemical Co. Osaka, Japan) for 30 min. Stained cells were visualized using a CKX31 microscope (Olympus Corp., Tokyo, Japan), and the positive area was calculated using ImageJ software (NIH, Bethesda, MD).

### Statistical Analysis

Statistical analysis measured mean and standard deviation (SD) by 3–5 independent studies. The statistical significance of the relation between groups by the F-test and the t-test was shown by the variance ratio test. There was a substantial difference when the risk rate was *p* < 0.05.
